# Feasibility, acceptability, and characteristics associated with adherence and completion of a culturally relevant internet-enhanced physical activity pilot intervention for overweight and obese young adult African American women enrolled in college

**DOI:** 10.1186/s13104-015-1159-z

**Published:** 2015-06-02

**Authors:** Rodney P Joseph, Gareth R Dutton, Andrea Cherrington, Kevin Fontaine, Monica Baskin, Krista Casazza, Danielle Lorch, Jeroan J Allison, Nefertiti H Durant

**Affiliations:** College of Nursing and Health Innovation, Arizona State University, 500 N. 3rd Street, Phoenix, AZ 85004 USA; Division of Preventive Medicine, University of Alabama at Birmingham, 1717 11th Avenue South, Birmingham, AL 35205 USA; Department of Health Behavior, School of Public Health, University of Alabama at Birmingham, 1665 University Blvd., Birmingham, AL 35293 USA; Department of Nutrition Sciences, School of Health Professions, University of Alabama at Birmingham, 1675 University Blvd, Birmingham, AL 35294 USA; Department of Psychology, University of Alabama at Birmingham, 1300 University Blvd, Birmingham, AL 35294 USA; Division of Health Informatics and Implementation Science, Department of Health Sciences, University of Massachusetts Medical School, 55 Lake Avenue North, Worcester, MA 01655 USA; Department of Pediatrics, University of Alabama at Birmingham, CPPI, Suite 410, 1600 7th Avenue South, Birmingham, AL 35233 USA

**Keywords:** Website, Black, Women, Exercise, Physical activity, Overweight, Obese, College, University

## Abstract

**Background:**

African American women are one of the least active demographic groups in the US, with only 36% meeting the national physical activity recommendations in comparison to 46% of White women. Physical activity begins to decline in African American women in adolescence and continues to decline into young adulthood. Yet, few interventions have been developed to promote physical activity in African American women during this critical period of life. The purpose of this article was to evaluate the acceptability and feasibility of a culturally-relevant Internet-enhanced physical activity pilot intervention for overweight/obese African American college females and to examine psychosocial and behavioral characteristics associated with intervention adherence and completion.

**Methods:**

A 6-month single group pre-posttest design was used. Participants (n = 27) accessed a culturally-relevant Social Cognitive Theory-based physical activity promotion website while engaging in a minimum of four moderate-intensity physical activity sessions each week. Acceptability and feasibility of the intervention was assessed by participant retention and a consumer satisfaction survey completed by participants.

**Results:**

Fifty-six percent of participants (n = 15) completed the intervention. Study completers were more physically active at baseline (P = 0.05) and had greater social support for exercise from family members (P = 0.04). Sixty percent of study completers (n = 9) reported the website as “enjoyable” or “very enjoyable” to use and 60% (n = 9) reported increased motivation from participation in the physical activity program. Moreover, 87% (n = 13) reported they would recommend the website to a friend.

**Conclusions:**

Results provide some preliminary support for the acceptability and feasibility of an Internet-enhanced physical activity program for overweight/obese African American women, while highlighting important limitations of the approach. Successful promotion of physical activity in college aged African American women as they emerge into adulthood may result in the development of life-long healthy physical activity patterns which may ultimately reduce physical activity-related health disparities in this high risk underserved population. Future studies with larger samples are needed to further explore the use of Internet-based programs to promote physical activity in this population.

## Background

The benefits of physical activity (PA) are well-established for the prevention and treatment of various chronic diseases such as cardiovascular disease, diabetes, obesity, and some cancers [1, 2]. However, despite these benefits, PA levels among minorities and women, particularly African American women, remain at levels substantially below recommended frequency and dose. National statistics show that only 36% of African American women achieve at least 150 min of moderate-intensity PA per week as recommended by US Department of Health and Human Services [3]. This is in comparison to 46% of White women [3]. The limited participation in PA among African American women is concerning given the benefits of PA and that these women are disproportionately burdened by the aforementioned chronic disease conditions [4]. Moreover, a staggering 80% of young adult African American women aged 20–39 are overweight or obese and 56% are obese (compared to 59% and 32% of non-Hispanic White women, respectively) [5]. Engagement in PA begins to decline in all girls during puberty [6, 7]. However, among African American females in particular, the most rapid reduction occurs in late adolescence [7, 8]. Thus, innovative methods to increase participation in PA programs are needed, particularly among African American women.

Despite the critical need for effective interventions to promote PA among young adult African American women, few interventions have addressed this group. One potentially promising mode to deliver PA interventions to young adult African American women is the Internet. Internet interventions have not only dramatically increased over the past decade but have shown positive outcomes for increasing PA [9–11]. However, among the studies conducted, few have included racially or ethnically diverse samples and most have included middle-aged adults (i.e. >40 years) [9, 10].

Our review of the English-language literature revealed that our research team is the only group that has published studies using the Internet to deliver culturally-relevant PA interventions to African American women [12, 13]. Given the paucity of research on this topic, the purpose of this article is to expand on the previously published outcomes [12] of a culturally-relevant Internet-enhanced PA promotion program for young adult overweight and obese African American women by: (a) examining psychosocial and behavioral characteristics associated with intervention adherence and completion, and (b) reporting outcomes associated with acceptability and feasibility of the Internet-enhanced approach. Select behavioral and psychosocial outcomes of the 6-month PA intervention are also briefly presented to provide readers with a general overview of primary study findings. Results provide preliminary insight regarding the use of the Internet to promote PA among African American women.

## Methods

### Intervention description overview: a internet-enhanced physical activity program

The PA promotion program used in the current study was a product of two formative research phases. In the first phase of study development, cognitive interviews and focus groups were conducted with the target population to identify website-based features young African American women desire in an Internet-enhanced PA program [14]. Data collected during this phase were used to inform development of the initial prototype of the study website. In the second phase of study development, a 6 week feasibility assessment of the intervention was implemented. This phase consisted of participants utilizing the study website as a PA promotion tool while engaging in supervised moderate-intensity PA sessions. Participants also participated in bi-weekly focus group over this 6 week period to provide feedback for website refinement. Data collected from this phase were used to finalize the website and study protocol implemented in the current study.

Young women enrolled in the current study agreed to use a culturally-relevant Internet-based application as a PA monitoring and promotion tool and participate in four 60 min moderate-intensity walking/exercise sessions per week for 6 months. Each of these intervention components is briefly described below; however, readers are referred to additional text for more detailed information regarding the study components and the primary study outcomes [11].

#### Website component

For the website component of the program, participants were asked to log-on to the study website a minimum of four times per week after completing their supervised PA sessions. The study website was informed by formative research with our target population [14] and included the following key features: (1) personal profile pages that allowed participants to upload photos and share personal information about themselves, (2) PA tracking tools, (3) weight and Body Mass Index (BMI) tracking tools, (4) motivational tools (including motivational quotes and forums such as message boards and blogs for participants to give testimonials regarding successful strategies for increasing PA), (5) exercise demonstration videos, and (6) electronic food diaries with the capability to allow participants to search and enter caloric and other dietary information on items consumed in a given meal or day. Cultural relevance of the website was achieved by: (a) including pictures of overweight and obese African American women throughout the study website, (b) creating an exclusive online environment for African American women to engage in dialogue associated with PA engagement, (c) including information regarding the low PA levels among African American women and associated chronic disease burden, (d) providing hair care tips for African American women, and (e) including motivational and inspirational quotes from prominent African American celebrities and historical African American community leaders.

#### Exercise component

The goal of the four exercise sessions was to have participants accumulate a minimum of 150 min of moderate-intensity PA per week as recommended by the US Department of Health and Human Services [1]. To meet this PA requirement, participants were encouraged to participate in supervised group walking sessions held at the university’s recreation center. Participants were also allowed to substitute one of their group walking sessions each week with a group-based exercise class (e.g. Zumba, kick boxing) sponsored by the recreation center. Supervised exercise sessions were offered during the morning and evening hours for participants to attend at their convenience. Morning exercise sessions were offered Monday through Friday from 6:00 a.m. to 9:00 a.m. and evening exercise sessions were offered Monday through Thursday from 4:00 p.m. to 8:00 p.m.

Participants wore pedometers and heart rate monitors to self-monitor the intensity and duration of their PA. Physical activity sessions were supervised by a trained research assistant. The role of the research assistant was to give instruction on use of heart rate monitors and pedometers and assist with interpretation of data from those devices. For group activities other than walking, participants wore study heart rate monitors and pedometers that they checked-out from study staff prior to the exercise session and returned to study staff to document the duration and intensity of the session.

### Participant recruitment

Participants were recruited via convenience sampling methods at the University of Alabama at Birmingham (UAB) during the spring 2011 semester. Interested participants completed a telephone screening to determine study eligibility. To be eligible participants had to: (a) be aged 19–30 years at time of enrollment, (b) have a BMI greater than or equal 25, (c) self-identify as African American, (d) be currently enrolled as an undergraduate or graduate student at UAB, and (e) have no self-reported medical conditions that would inhibit or limit performance of PA. Individuals were excluded if they reported: (a) participating in another PA, nutrition, or weight loss program at the time of the study enrollment, (b) current use of weight loss medications, (c) weight loss of greater than 10 pounds in the 3 months prior to the study enrollment, (d) history bariatric surgery, or (e) uncontrolled high blood pressure (defined as greater than 140/90) at the time of the study enrollment. The study was approved by the UAB Institutional Review Board and each participant signed informed consent. Participants were eligible to receive a maximum of $150 compensation for completing the study.

### Outcome measures

#### BMI

Weight and height measures were collected by trained study staff in order to calculate BMI. Weights were measured to the nearest kilogram using a Scaletronix (Wheaton, Il, USA) digital scale. Height was measured to the nearest inch using the Digi-kit stadiometer by Measurement Concepts and Quick Medical (North Bend, WA, USA). To ensure consistency of height measurements, the same research staff member assessed height for all participants.

#### Physical activity

Physical activity was assessed by the Seven Day Physical Activity Recall (7-Day PAR) [15]. The 7-Day PAR is an interviewer-administered questionnaire that utilizes a standardized, semi-structured interview to assess duration, intensity, and frequency of PA [16]. The standardized interview process used by the 7-Day PAR has demonstrated significant test–retest reliability estimates among adolescent (r = 0.81) [17] and young adult populations (r = 0.99) [18], and has strong inter-rater reliability (r = 0.78) for assessments performed by multiple interviewers with the same subject [15]. The recall instrument has been used to assess PA among AA in various studies [19, 20] and has been validated against more objective measures of PA such as doubly labeled water [21], PA logs [22], and accelerometers [23].

#### Social support

Social support for exercise was evaluated using the Social Support for Exercise Survey [24]. This scale assesses two separate types of social support, family support (13-items) and support from friends (10-items). The Social Support for Exercise Survey has demonstrated adequate test–retest reliability (0.79 and 0.77 for the family and friends scales respectively, p < 0.0001) [24] and has internal consistency (Cronbach alphas) estimates ranging from 0.88 to 0.91 when used exclusively in African American populations [25]. This survey demonstrated internal consistency estimates ranging from 0.82 to 0.91 for the family scale and 0.91 to 0.95 for the friends scale in the current study.

#### Self-efficacy

The Exercise Confidence Survey [26] was used to assess exercise self-efficacy. This 12-item survey asks respondents to indicate how confident they are in their ability to consistently motivate themselves to do a series of exercise-related activities over the next 6 months. This survey has previously established reliability and validity [26] and demonstrated internal consistencies ranging between 0.84 and 0.94 in the current study.

#### Website usage

Website utilization was assessed by an algorithm employed by the study website. Each time a participant logged onto the website and engaged in using an application, the algorithm calculated a number of points based on the amount of time spent on the website and the number and type of applications used. Therefore, the more frequently participants logged onto the website and used the available applications, the higher the number of points they accumulated over the course of the study. Table 1 illustrates how points were awarded according to website usage.

**Table 1:** Overview of the point algorithm for website utilization

Website activity	Assigned point value
Submitting workout on the activity tracker	10
Updating personal weight	10
Updating body measurements (waist, hips, thigh, etc.)	2
Updating personal profile status	5
Uploading a profile picture	50
Requesting to be friends with another user	1
Posting on another user’s wall	2
Commenting on another user’s wall post	1
Joining a group	2
Posting on a group’s wall	2
Posting on a challenge’s wall	2
Replying to a message board thread	2
Having other users reply to message board thread you created	2
Setting-up a personal blog	10
Commenting on an exercise, workout plan or diet plan	2

#### Satisfaction measure

At the conclusion of the intervention, participants completed a consumer satisfaction survey. This survey was adapted from previous research [19, 27] and designed to assess the acceptability and feasibility of the Internet-enhanced approach. Example items from this questionnaire include: “Overall, how helpful do you find the study website?” and ‘Would you recommend the study to a friend?” Only participants completing the 6-month study completed the satisfaction measure as this survey was completed at the study’s final follow-up assessment.

### Procedures

At the baseline study visit, participants provided informed consent and completed demographic and psychosocial (i.e., self-efficacy and social support) questionnaires. Height and weight were also measured and participants completed the 7-Day PAR [15]. Similar data collection procedures were conducted at the midpoint (3-month) and final follow-up assessments (6-months).

### Statistical analyses

Normality of the data was assessed using box-plots, histograms and QQPlots. Despite the small sample size, all outcome measures met assumptions for normality with the exception of PA. Independent *t*-tests were used to compare characteristics associated with intervention adherence and completion. Specifically, in a first set of analyses, we examined whether baseline characteristics of BMI, PA, and various psychosocial variables differed among participants who completed the intervention versus those who did not complete the intervention. In a second set of analyses, we explored whether baseline characteristics of women who were more adherent to the recommended group-based exercise sessions differed from women who were less adherent. For this analysis, we dichotomized women as either engaging in 55% or more of the group-based exercise sessions or engaging in less than 55% of the recommended exercise sessions. The cut-point of 55% was selected because this was median percent of session attendance among study completers. Paired *t*-tests were used assess pre-post changes in BMI, social support, and self-efficacy. Wilcoxon signed rank tests were used to assess changes in PA. Statistical significance was set a *P* ≤ 0.05.

## Results

### Participants

**Figure 1:**
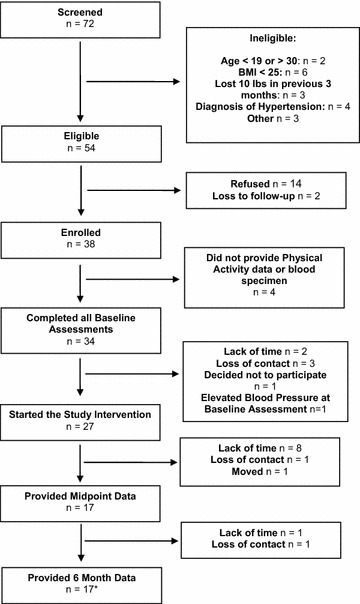
Participant flow. *Asterisk* includes two participants that did not provide midpoint data.

Figure 1 illustrates the participant flow diagram. Seventy-two African American females were screened for eligibility. Of these, 54 were considered eligible and 38 provided informed consent to participate. Thirty-four of the 38 (89%) consented individuals completed the baseline assessment required to participate in the intervention. Of the 34 women who completed the baseline assessment, 7 failed to start the intervention component. Reasons given for participant withdrawal between the baseline assessment and the start of the intervention included lack of time due to work and school schedules (n = 2), and “concern” for lack of enjoyment during the intervention (n = 1). Additionally, three participants were lost to follow-up between the assessment and the start of the intervention component and one participant was excluded due to elevated blood pressure at the baseline assessment.

Twenty-seven women started the Internet-enhanced PA program. Fifteen of the 27 (56%) participants who began the web enhanced PA program met criteria for completion of the study program; criteria for completion was defined as providing data at all three assessment periods (baseline, midpoint, 6 months).

At baseline (see Table 3), participants (n = 34) had a mean age of 21.21 years (*SD* = 2.29) and were mostly obese (*M* BMI = 35.37, *SD* = 6.82). The majority were never married and seeking an undergraduate degree. Participants reported low levels of baseline PA as assessed by the 7-Day PAR (*M* = 81.76, *SD* = 76.28) and relatively high levels of the related psychosocial variables.

### Exercise session attendance and website usage

Table 2 illustrates exercise session adherence and website usage points for participants who began the Internet-enhanced program. Among the 27 women who started the PA intervention, median PA session attendance was 42.1% (range 2.9–84.6%) and the median number of website usage points was 56.00 (range 0–1,726). Women who completed the intervention (n = 15) attended a median of 54.92% of PA sessions (range 13.5–84.6%) and had a median of 130.00 website points (range 10.00–1,726.00). Participants who did not complete the intervention attended a median of 9.61% (range 2.9–59.6%) of exercise sessions and had a median of 11.00 (range 0.00–1,531.00) website points.

**Table 2:** Exercise session adherence and website usage points for participants (n = 27) who began the Internet-enhanced physical activity program

	Percent exercise session attendance Median (range)	Website usage points Median (range)
All participants (n = 27)*	42.1 (2.9–84.6)	56 (0–1,726)
Completers (n = 15)	54.92 (13.5–84.6)	130 (10–1,726)
Non-completers (n = 12)	9.6 (2.9–59.6)	11 (0–1,531)

* Includes only the 27 women who began the Internet-enhanced program.

### Characteristics associated with intervention completion and adherence

Table 3 illustrates participant characteristics associated with intervention adherence and completion. Comparative analysis revealed that women who completed the study were more physically active at baseline (*P* = 0.05) and had greater family social support for exercise (*P* = 0.04). Conversely, there were no significant baseline differences among women who completed at least 55% of the supervised exercise sessions versus those completed less than 55% of the exercise sessions.

**Table 3:** Baseline characteristics of enrolled participants that provided complete baseline data (N = 34)

Variable	All participants (N = 34)	Completers (N = 15)	Non-completers (N = 19	Completers vs. non-completers (*p* value)	Attended ≥55% of exercise sessions (N = 8)	Attended <55% of exercise sessions (N = 26)	Attended ≥55% vs. <55% of exercise sessions (*p* value)
Age, mean (SD)	21.12 (2.29)	21.87 (2.70)	20.53 (1.81)	0.09	21.25 (2.32)	21.08 (2.33)	0.86
Marital status, n							
Never married	31	13	18	N/A	7	24	N/A
Married	1	0	1		0	1	
Divorced	1	1	0		0	1	
No answer	1	1	0		1	0	
Degree currently obtaining; n							
Undergraduate	32	13	19	N/A	6	19	N/A
Masters	1	1	0		1	0	
Doctoral	1	1	0		1	0	
BMI, mean (SD)	35.37 (6.82)	35.60 (8.11)	35.20 (5.83)	0.87	34.63 (4.14)	35.60 (7.51)	0.73
Physical activity, mean (SD) (min/week)^a^	81.76 (76.28)	111.00 (88.89)	58.69 (56.98)	0.05	124.38 (109.76)	68.65 (59.56)	0.21
Self-efficacy, mean (SD)	3.97 (0.57)	4.05 (0.57)	3.90 (0.59)	0.47	4.09 (0.44)	3.93 (0.61)	0.51
Social support from Friends^b^, mean (SD)	2.74 (1.00)	2.74 (0.93)	2.74 (1.07)	0.99	2.88 (0.99)	2.70 (1.01)	0.30
Social support from Family^b^, mean (SD)	2.20 (0.86)	2.53 (0.84)	1.92 (0.79)	0.04	2.47 (0.75)	2.11 (0.89)	0.66

Completers were defined as providing data at all three assessment periods (baseline, midpoint, 6 months), logging onto the study website a minimum of once, and attending at least one exercise session. Comparative analysis for variables of marital status and degree currently seeking were not conducted because the majority of participants (95%, n = 32) were single and enrolled as undergraduates.

^a^Physical activity assessed by the Seven Day Physical Activity Recall.

^b^Mean survey scores.

### Physical activity and psychosocial outcomes

Study outcomes for PA, BMI, self-efficacy and social support are reported in Table 4. At the midpoint assessment (3 months), study completers significantly increased their PA by an average of 74 min/week (*SD* = 125.28; Wilcoxon *Z* = 2.05, *P* = 0.04) and reported a significant decrease in exercise self-efficacy (*P* = 0.03). At 6 months, PA subsequently declined from the midpoint resulting in a non-significant increase from baseline to 6 months (Wilcoxon *Z* = 0.68, *P* = 0.50). Social support from friends significantly increased from baseline to 6 months (*P* = 0.02) and BMI remained stable throughout the intervention. No pre-post intervention changes in social support from family or exercise self-efficacy were observed.

**Table 4:** Outcomes at 3 and 6 months for overweight and obese African American women who completed a 6 month web enhanced physical activity program, n = 15

Variable	Means (SD)			Mean difference (SD)			
	Baseline	3 months	6 months	Base to 3 months	*p*	Base to 6 months	*p*
BMI	36.00 (8.26)	36.03 (8.64)	36.14 (9.17)	0.03 (0.57)	0.86	0.14 (1.13)	0.64
Physical activity (min/week)	111.00 (88.87)	184.93 (96.56)	136.00 (97.70)	73.93 (125.28)	0.04^a^	25.00 (93.21)	0.50^a^
Social support from family	2.53 (0.84)	2.44 (0.91)	2.50 (0.53)	−0.10 (0.84)	0.66	−0.03 (0.68)	0.86
Social support from friends	2.74 (0.92)	3.04 (1.16)	3.14 (0.99)	0.30 (0.91)	0.26	0.40 (0.60)	0.02
Exercise self-efficacy	4.05 (0.57)	3.68 (0.60)	3.80 (0.87)	−0.37 (0.57)	0.03	−0.25 (0.69)	0.18

^a^ *p* value using Wilcoxon signed rank test.

### Feasibility and acceptability

Participants who completed the intervention were largely supportive of the Internet-enhanced PA program. Sixty percent (n = 9) reported the website as “enjoyable” or “very enjoyable” and 20% (n = 3) responded it was “somewhat enjoyable.” Additionally, 60% (n = 9) reported increased motivation from participation in the program. Among the applications available on the website, participants self-reported using the PA tracker application most frequently followed by the diet tracker application, exercise demonstration videos, message boards and wall posts. The website’s analytical tracking software corroborated these self-reported findings. Participants also reported that their preferred modality to receive Internet-enhanced PA promotion programs was via a Smartphone. Moreover, 87% (n = 13) reported they would recommend the website to a friend.

Most participants responded favorably to the group exercise sessions. Among those who completed the intervention, 73.3% (n = 11) identified them as “helpful” or “very helpful” for promoting PA. As well, 93% (n = 14) of participants indicated that the walking sessions were “somewhat enjoyable” to “very enjoyable”.

## Discussion

The purpose of the this study was to examine psychosocial and behavioral characteristics associated with completion and adherence to a culturally relevant Internet-enhanced PA program specifically *designed* to promote PA in overweight and obese young adult African American enrolled in college and to assess the acceptability and feasibility of the approach. Young adult African American women represent an underserved population with respect to PA promotion [28–30]. While participant retention was modest, results for study completers showed a statistically significant increase in PA at 3 months. At 6 months, however, PA attenuated and was no longer greater than baseline. The initial increase and subsequent decline in PA over the duration of the study was somewhat unexpected and emphasizes the need for identification of effective strategies promote long-term maintenance of high PA levels among African American women. We hypothesize that the initial increase and subsequent decline in PA was associated with participants attending supervised exercise sessions at the 3-month assessment and the fact that these sessions were no longer offered during the 6-month assessment (as the intervention was completed). We also observed enhanced social support from friends from baseline to 6 months among participants who completed the intervention, in addition to high participant satisfaction with the study website. Results of this exploratory pilot provide some promising findings of the Internet-enhanced approach and offers important insight for future work with this population.

Results showed that participants with higher levels PA at baseline were more likely to complete the intervention. This finding indicates the need to further refine and tailor the protocol to identify ways to improve adherence among participants with the lowest levels of PA at study enrollment. However, we note that this outcome is similar to findings reported by Stephen et al. [31] which found that adults with previous exercise experience had higher adherence to a moderate-intensity exercise program (i.e., 65–75% of the maximum heart rate reserve) than participants with limited past exercise experience. A potential strategy to improve adherence among participants with low PA levels at baseline could include scheduling these participants to attend group exercise sessions together. This would provide more tailored approach and allow less fit individuals to work together and gradually reach their PA goals.

Women who completed the intervention also reported higher levels of family support for PA. We did not include a family component in this intervention. However, involvement of family, particularly for individuals with lower PA levels may increase retention. While prior studies in older African American women have cited family as a barrier to PA [32], family may operate differently in younger African American women. Future research in this population should explore the role of family and the potential for the inclusion of family in the promotion of PA in young adult African American women.

We were surprised to discover that baseline characteristics of women who were more adherent to attending the exercise session component of the PA program (i.e. attended ≥55% of exercise sessions) did not differ from women who were less adherent. This unexpected outcome may be associated with insufficient statistical power due to the small sample of our study (especially given differences in PA levels between these two groups). Nonetheless, the low exercise session attendance among participants indicates the need for additional research to explore strategies to improve adherence to this component of the program. Feedback from both women who completed the study and withdrew indicated that the allocated times for the supervised exercise sessions (i.e. from 7 a.m. to 9 a.m. Monday through Friday and 4 p.m. to 8 p.m. Monday through Thursday) were not conducive to their work and/or class schedules. Participants indicated the desire to exercise during the day between classes and/or later at night. Future studies should take this information into consideration when designing PA programs African American women enrolled in college and provide available times for participants to engage in supervised PA throughout the day.

We also observed a decrease in self-efficacy between baseline and 3 months, suggesting the need for future work to study how to sustain self-efficacy for PA. It is possible that the participants’ initial success in PA during the first 3 months of the study and no observed decrement in BMI was disappointing for participants, which lead to both a decline in self-efficacy of PA and ultimately a decline in PA at 6 months after the initial increase at 3 months. This information is enlightening and can be used to guide future intervention efforts. Specifically, futures studies should address how low or modest success during initial attempts at increasing PA may preclude motivation and thus decrease PA over the course of the intervention.

Program satisfaction was high among completers with the majority of participants endorsing the website as a source of enjoyment and motivation to exercise. Moreover, over 80% of participants who completed the intervention reported they would recommend the website to a friend. These data show some promise for using a culturally relevant website to promote participation in PA in this high risk, understudied population. However, this positive feedback must be considered in the context of several limitations.

While women who completed the study endorsed enjoyment and use of the Internet-based tool, the low website usage among participants tempers these favorable self-report findings. We note though, that low website usage is a commonly reported issue among Internet-based PA programs [9]. Feedback from participants indicated that reasons for low website usage included lack of time to log-on to the study website and that the study website was not as user-friendly as they had expected (i.e., had too many features and was difficult to navigate). These findings suggest the need to make the study website more easily accessible (eg., through a mobile phone website platform or Smartphone Application) and user-friendly. The modest retention (56%) and lack of formal post-intervention feedback from women who withdrew from the study were also limitations. Researcher should explore strategies to increase retention and PA engagement among African American women and attempt to obtain formal feedback from withdrawn participants to inform future research efforts. Some potential strategies to increase participant retention may include providing more frequent incentives or updating the study website with PA-related information more frequently. Additionally, we did not collect data regarding whether participants had children or on the intensity of their semester course load. Such information would have been beneficial to include when evaluating study outcomes and will be collected in future research. Other limitations include the small sample size and inclusion of only African American women enrolled in undergraduate and graduate classes. Due to these limitations, results may not be generalizable to those not enrolled in college.

## Conclusions

This exploratory study is an important first step in understanding the potential role of technology in the delivery of a culturally relevant web enhanced PA program to overweight and obese young adult African American women. Future studies are needed to explore the use of Internet and mobile phone technology to enhance the delivery of PA programs in this understudied population. We propose that future studies explore the use of cellular and Smartphone technology to enhance the delivery of PA promotion programs among young adult African American women, as participants indicated that they would prefer delivery of such programs via Smartphone. Likewise, findings of recent studies have shown favorable outcomes for the use of mobile phone technology to promote PA [33–37]. However, the majority of interventions conducted have focused on middle and older aged adults [35, 36]; indicating the need for additional research on how this technology can be used to promote PA in young adult African American women. There is also a need to design and conduct randomized controlled trials to assess the role of technology in the promotion of PA in overweight and obese young adult African American women. The integration of culturally relevant based PA promotion interventions that leverage state of the art technology may be key in combating the obesity epidemic in this high risk, understudied population.

## Abbreviations

PA: physical activity
UAB: University of Alabama at Birmingham
BMI: Body Mass Index
7-Day PAR: Seven Day Physical Activity Recall
